# Cost-effectiveness analysis of an innovative model of care for chronic wounds patients

**DOI:** 10.1371/journal.pone.0212366

**Published:** 2019-03-06

**Authors:** David Brain, Ruth Tulleners, Xing Lee, Qinglu Cheng, Nicholas Graves, Rosana Pacella

**Affiliations:** 1 Australian Centre for Health Services Innovation (AusHSI), Brisbane, Queensland, Australia; 2 Institute of Health and Biomedical Innovation, Queensland University of Technology, Kelvin Grove, Queensland, Australia; 3 Wound Management Innovations Cooperative Research Centre, Brisbane, Australia; 4 The University of Chichester, West Sussex, United Kingdom; Medical University of Graz, AUSTRIA

## Abstract

Current provision of services for the care of chronic wounds in Australia is disjointed and costly. There is large variability in the way that services are provided, and little evidence regarding the cost-effectiveness of a specialist model of care for treatment and management. A decision-analytic model to evaluate the cost-effectiveness of a specialist wound care clinic as compared to usual care for chronic wounds is presented. We use retrospective and prospective data from a cohort of patients as well as information from administrative databases and published literature. Our results show specialist wound clinics are cost-effective for the management of chronic wounds. On average, specialist clinics were $3,947 cheaper than usual clinics and resulted in a quality adjusted life year gain of 0.04 per patient, per year. Specialist clinics were the best option under multiple scenarios including a different cost perspective and when the cost of a hospital admission was reduced. Current models of care are inefficient and represent low value care, and specialist wound clinics represent a good investment compared to current approaches for the management of chronic wounds in Australia.

## Introduction

Despite emerging evidence that best practice wound care coincides with health improvements and cost savings, the majority of Australians with chronic wounds do not receive best practice care [1–6]. Services for those not receiving best practice care are fragmented, with care delivered by a range of healthcare professionals across primary and secondary settings [1]. Such a disconnected system leads to increased healing times, high recurrence rates, complications requiring hospitalisation and reduced quality of life for the thousands of patients affected by chronic wounds in Australia [7, 8]. Partly due to a lack of reimbursement for wound services and consumables in the Australian setting there are avoidable costs for both patients and the Australian health system [7]. Internationally, best practice models of care for chronic wounds mostly evaluate patient health outcomes, with some studies reporting the associated costs [9–11], however, the majority do not conclude in a detailed cost-effectiveness analysis. If economic evaluations are performed, they tend to focus on specific wound types or treatments, rather than assessing a holistic change in practice. For example, economic evaluation of guideline based treatment for diabetic foot ulcers [12]; or compression therapy for venous leg ulcers exist [13], but these studies do not assess a new model of care, from an economic perspective. Whether widespread implementation of an innovative specialist model of care for wound treatment and management provides good value for money in the Australian setting is not known. Therefore the aim of this research is to present an economic evaluation of a specialist wound service, to estimate the cost-effectiveness of an innovative approach to service delivery, compared to usual care.

## Methods

This evaluation follows the guidelines presented in the *Consolidated Health Economic Evaluation Reporting Standards* (CHEERS) checklist [14]. Following this checklist is recommended to ensure consistency and rigour when reporting economic findings. The completed checklist can be found in S1 Appendix.

### Description of service delivery

#### Specialist wound clinics

A specialist wound care service was opened in Brisbane on 30 January, 2017. Patients referred to the clinic are seen by a multidisciplinary team, comprising of a vascular specialist, a wound nurse practitioner candidate, an advanced clinical podiatrist, and a registered nurse. Patients are provided with tailored wound dressing plans, designed to be managed by the patient or primary carer outside the clinic, at future clinic visits, or augmented through telehealth. Patients can access the specialist clinic through referrals from their primary care provider, but are also able to self-refer. Referrers receive a report outlining the diagnosis, treatment provided and recommendations for follow up care. Most patients admitted to the clinic have received some care for their wound prior to attendance, most commonly from their general practitioner (GP). Telehealth services are also available to patients, where a real-time video consultation between health professionals, the clinical team and the patient, takes place. This service is designed to reach those who may not be able to attend clinic appointments in person, for example, those who live in rural or remote settings, or those in residential aged care facilities. Consultations are attended by one or more specialists, and a detailed treatment plan is created and reviewed by the patient and their primary carer.

#### Usual care (historical comparator)

Due to the disjointed nature of service delivery, patients in the Australian setting are likely to access a large number of healthcare providers for wound treatment, often using a combination of primary and secondary services. General practitioners are most frequently utilised, either on their own or in combination with allied health support, community nursing staff, or with medical specialists. Due to the lack of specialist wound management training [15, 16] the majority of these service providers are unlikely to be in a position to provide patients with coordinated, best practice care. The number of appointments attended and services accessed varies considerably between patients, due to factors related to patient or carer education, mobility and capacity to pay for services. Given this significant variation in accessing practices, healthcare utilisation for the 12 month period prior to enrolment at the specialist clinic was collected from patients enrolled in the study. This information, coupled with data collected from Medicare Benefits Schedule (MBS) and Pharmaceutical Benefits Scheme (PBS) about service utilisation, was used to characterise ‘usual care’ for this evaluation.

### Study design

Demographic information, medical history, risk factors, clinical assessment of wounds and quality of life data were collected from patients upon enrolment in the study, using clinical records and patient interview. All patients provided retrospective data for the previous 12 months, relating to healthcare utilisation, procedures received, investigations undertaken, hospital admissions, wound-related complications, services accessed, healing rate, dressings used and weekly out of pocket costs. This patient-reported data was validated through a review of clinical records and relevant MBS and PBS data.

The same data were collected prospectively for 3 months following enrolment in a specialist wound clinic. Descriptive data from this cohort has been published elsewhere [17]. Data relating to participants’ healthcare utilisation and pharmaceutical usage was retrieved from the MBS and PBS databases. The MBS and PBS data were accessed for the 12 months prior to the study starting and for the 3-month follow up period, after enrolment, and was cross-checked with patient self-reported information. Ethical approval for the study was granted by the Metro South Human Research Ethics Committee, reference number HREC/17/QPAH/363.

### Decision-analytic model

**Fig 1:**
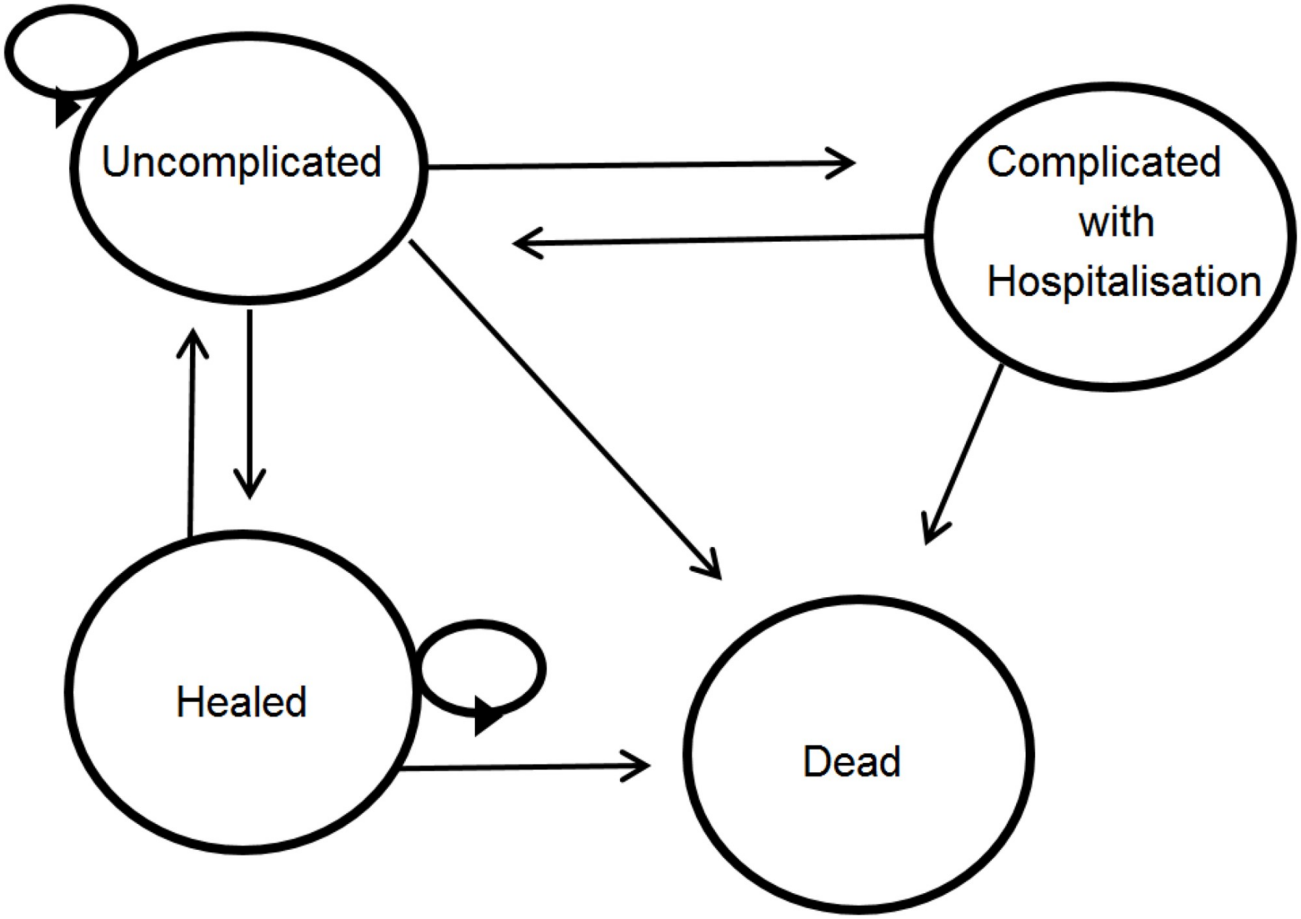
Pictorial representation of the Markov model used for economic evaluation of specialist wound care clinic.

A decision-analytic model was developed to evaluate the cost-effectiveness of the specialist wound care clinic compared to usual care. Given the chronic and repetitive nature of wounds, the model needed to be state-based and able to include recursive events, so a Markov model was chosen [18]. A pictorial representation of the model is shown in Fig 1. The model depicts the possible movement of patients through its health states over time, with each health state having a cost and a health utility weight attached to time spent in that state. This structure provides the framework for the evaluation and is used to estimate the costs and health outcomes associated with the differing approaches to service provision.

The model contains four health states; ‘uncomplicated’, ‘complicated with hospitalisation’, ‘healed’ and an absorbing state, ‘dead’. The model’s cycle length was 1 month, and it was run for 12 cycles to complete a 1-year time horizon. The modelled patient cohort was a 50:50 ratio of male and female patients, with an average age of 72 years. This demographic was chosen to reflect the patient cohort from whom data were collected in the study [17]. Further information on patient demographics are shown in Table 1.

**Table 1:** Baseline demographic, health and wound characteristics.

Characteristics	Total n = 29 (Wound type n = 30)*	Venous leg/ Gravitational Ulcers n = 22 73%	Diabetic Foot Ulcers n = 2 7%	Other** n = 6 20%
**Demographic**				
Age (mean, range)	72 (25–92)	75 (25–92)	63 (45–81)	69 (51–88)
**Gender**				
Male [n (%)]	13 (45%)	9 (41%)	1 (50%)	3 (50%)
**Co-morbidities/Health**				
Body Mass Index (mean, range)	28.32 (17.3–47.4)	27.68 (17.3–47.4)	35.75 (30.5–41)	28.36 (24.9–33.7)
Diabetes mellitus [n (%)]	5 (17%)	3 (14%)	2 (100%)	1 (17%)
Autoimmune disorders [n (%)]	4 (14%)	2 (9%)	0 (0%)	2 (33%)
Haematological disorders [n (%)]	5 (17%)	2 (9%)	0 (0%)	3 (50%)
Smoker [n (%)]	1 (3%)	0 (0%)	0 (0%)	1 (17%)
Previous smoker [n (%)]	5 (17%)	4 (18%)	0 (0%)	1 (17%)
Hypertension [n (%)]	19 (66%)	15 (68%)	2 (100%)	3 (50%)
Venous insufficiency [n (%)]	13 (45%)	12 (55%)	0 (0%)	1 (17%)
Previous amputation [n (%)]	2 (7%)	2 (9%)	0 (0%)	0 (0%)
**Wound characteristics on admission**				
Recurrence				
New Recurrent [n (%)]	14 (48%)	9 (41%)	1 (50%)	4 (67%)
New Not Recurrent [n (%)]	13 (45%)	11 (50%)	1 (50%)	2 (33%)
Healed with history of previous ulceration*** [n (%)]	2 (7%)	2 (9%)	0 (0%)	0 (0%)
**Baseline measurements**				
Wound area cm^2^ (mean, range)	18.15cm^2^ (0.1–184.4)	24.78 cm^2^ (0.1–184.4)	7.55cm^2^ (0.1–15)	8.66cm^2^ (0.5–31.8)
Wound duration—weeks (mean, range)	96 (8–480)	125 (8–480)	20 (8–32)	24 (8–48)

*1 participant had two wound types– 1 diabetic foot ulcer and 2 venous/gravitational ulcers. This participant was therefore included in both wound types

**Other wounds were scar breakdown, mixed venous/arterial, surgical dehiscence, pyoderma gangenosum vasculitic ulcer

***2 participants were receiving preventative treatment from the clinic to reduce the chance of recurrence. Neither participant had presented at the clinic with an active wound prior to treatment.

Because we are comparing the costs and health outcomes of treatments for patients with a wound, all patients in the modelled cohort began in the ‘uncomplicated’ health state. At each cycle of the model, patients in this state can either remain with an uncomplicated wound, die from other causes, enter the ‘healed’ state or have their condition worsen and move to the ‘complicated with hospitalisation’ state. Those patients in the ‘complicated with hospitalisation’ state could enter the ‘dead’ state or return to the ‘uncomplicated’ state, but they could not enter the ‘healed’ state directly after their hospitalisation. Given that on average patients are hospitalised for about a month because of their wound, patients could not remain in the hospitalised state for more than one cycle [18]. To reflect the true nature of wound epidemiology, patients who entered the ‘healed’ state are at risk of recurrence and could return to the ‘uncomplicated’ health state. Those who do not have a recurrence can either remain in the ‘healed’ state, or enter the ‘dead’ state, reflecting the possibility of death from all other causes. Patients under specialist care and usual care have different transition probabilities associated with their movement through the model. The model was constructed and analysed in Microsoft Excel 2010.

### Input data for the model

Movement between health states is based on transition probabilities, which were estimated from various sources shown in in Table 1. The cost and health outcomes associated with being in each health state were calculated per cycle and then summed over the 12 cycles of the model. Given that the model was run for 1 year, discounting was not applied to costs or health outcomes [19].

#### Transition probabilities

Where possible, we used data collected from the study’s patients to estimate transition probabilities for the model. For transitions that could not be estimated from primary data, such as dying from non-wound related causes, we used estimates that were published in the literature. Using data from a variety of sources to estimate transition probabilities is commonplace for this type of evaluation [20].

To estimate the probability of complication, where patients’ wounds deteriorate to the point of requiring hospitalisation, we used retrospective data from the trial cohort for usual care. To inform these estimates for specialist clinics, we prospectively collected data about wound-related hospitalisations for the cohort, in the trial period. For usual care, the same healing rate that was used in a previously published economic evaluation was used for this model [13]. For specialist clinics, we prospectively collected healing data from the trial cohort, where 45% (n = 9) achieved complete healing by the end of the data collection period. We used previously published estimates to estimate wound recurrence for usual care [13] and for specialist clinics [1]. All-cause mortality was calculated for the modelled cohort, using age and gender specific estimates from the Australian Bureau of Statistics [21]. To estimate death from wound-related causes, we used the all-cause rate, multiplied by the hazard ratio of dying from being hospitalised with a wound that was reported elsewhere [22]. We assumed no difference in all-cause, or wound-related mortality for patients receiving usual care or care from specialist clinics, in the model. Due to the model’s cycle length being 1-month, all probabilities were transformed to monthly probabilities, using a standardised formula [18]. The probability of remaining in a health state was simply calculated as 1 minus the sum of the other probabilities associated with leaving that health state [23]. Sources of evidence, and values used in the model are summarised in Table 2.

**Table 2:** Input variables for the Markov model.

Variable	Fixed Value	Distribution	Ref
***Transition Probabilities***^+^			
**Usual Care**			
Complication with hospitalisation (annual)	0.379	Beta	Study cohort
Healing rate (3-month)	0.228	Beta	[13]
Death (annual, all-cause)	0.016	Beta	[21]
Death (annual, wound related)	0.024	Beta	[12]
Recurrence (annual)	0.557	Beta	[13]
**Specialist Clinics**			
Complication with hospitalisation (3-month)	0.034	Beta	Study cohort
Healing rate (3-month)	0.345	Beta	Study cohort
Death (annual, all cause)	0.016	Beta	[21]
Death (annual, wound related)	0.024	Beta	[12]
Recurrence (annual)	0.222	Beta	[1]
Costs*	**AUD, monthly**		
**Usual Care**			
Uncomplicated	$521	Gamma	Study cohort
Complicated with hospitalisation	$30,319	Gamma	Study cohort
Healed	$68	Gamma	Study cohort
**Specialist Clinics**			
Uncomplicated	$1,169	Gamma	Study cohort
Complicated with hospitalisation	$30,319	Gamma	Study cohort
Healed	$84	Gamma	Study cohort
***Quality of life utility scores***			
Uncomplicated	0.69	Beta	Study cohort
Complicated with hospitalisation	0.59	Beta	Calculated
Healed	0.89	Beta	Study cohort

^+^Annual or 3-monthly probabilities were transformed to monthly probabilities using the formula: tp = 1-(1-tpt)^1/t^ [18]

*Costs have been described in full in appendix 2

#### Quality of life

Quality of life data were collected using the EuroQol-5D-5L tool and Visual Analogue Scale. Data were collected from participants upon enrolment, as well as after one and three months’ involvement in the study. Given that participants had current wounds on enrolment, we used baseline scores to estimate utility weights for the ‘uncomplicated’ health state. To capture the results of wound healing, scores at three months were used to estimate utility weights for the ‘healed’ state. These estimates were similar to utility weights published elsewhere [13, 24]. We were unable to estimate quality of life for patients who had been hospitalised, so we assumed a decrement of 0.1 utility from the uncomplicated state, to represent the ‘complicated with hospitalisation’ health state. This approach has been undertaken elsewhere [13], and is reported in the National Institute for Health and Care Excellence guidelines [25].

#### Costs

Costs associated with each health state were measured and valued in 2017 Australian dollars. We included patient out-of-pocket costs, such as travel/parking, dressing costs and clinic attendance, but we did not include costs associated with personal lost productivity due to time off work or other societal costs. Healthcare system costs, such as those associated with a hospital stay, medical appointment or prescriptions were obtained via multiple sources and included in the model. For costs associated with a hospital stay, we used estimates from a previously published economic evaluation, which valued a ward-bed at $800 per day [26]. As a result, our perspective is best described as a restricted societal approach to the analysis. A detailed breakdown of the costs included for each health state in the model is provided in S2 Appendix.

#### Model outputs

The main purpose of this evaluation is to estimate the expected value for money of specialist wound clinics, compared to usual care. This is indicated by the incremental cost-effectiveness ratio (ICER), where the mean change to costs associated with specialist clinics is divided by the mean change in quality adjusted life-years (QALYs) [27]. The ICER is compared against a cost-effectiveness threshold, which is assumed to be the willingness to pay (WTP) for an additional QALY, with ICERs that fall under the threshold deemed to be ‘cost-effective’. The threshold for this study is $64,000 (AUD), which is based on published estimates for the Australian setting [28].

#### Handling uncertainty

To quantify the impact of uncertainty relating to the model’s inputs on the model outputs, probabilistic sensitivity analysis was undertaken. The model was evaluated 10,000 times, using Monte Carlo simulations, with each simulation taking a random draw from each parameter’s distribution [20]. Transition probabilities and health utilities were assigned beta distributions, while costs were assigned gamma distributions, reflecting the skew usually associated with this type of data [20]. The change to costs and the change to QALYs was recorded for each model simulation, producing 10,000 pairs of incremental costs and effects. Practical issues with using the ICER for decision-making exist, given that the ratio of two numbers has awkward statistical properties [29]. In order to simplify this ratio information to a single number, the net monetary benefit (NMB) framework is used. Results are converted from the ICER to a NMB value, through the linear rearrangement of the ICER equation, as follows:

NMB=(WTPthreshold×ChangeinEffects)–ChangeinCosts

The interpretation of cost-effectiveness becomes particularly simple using the NMB: a positive NMB indicates that a strategy is cost-effective and a negative NMB indicates that a strategy is not cost-effective. Using the net benefit framework gives decision-makers a clear framework for choosing to adopt, or not adopt, the intervention being evaluated.

Uncertainties in other aspects of the evaluation also exist and were explored through scenario analyses. We changed key parameter values in the model to reflect plausible situations in the Australian setting, which not only tests the robustness of the model but also increases the breadth of information available to decision-makers. Four alternate scenarios were considered: (1) where only health system costs are included in the model and no patient out-of-pocket costs are included, (2) where only patient out of pocket costs are included in the model and health system costs are excluded, (3) the method for valuing hospital-bed costs is altered, where accounting costs for ward-beds is substituted by the lower price defined by a healthcare decision-maker’s willingness to pay for freed capacity [30], and (4) where the rate of healing from an uncomplicated wound was estimated in line with previously published literature (mean, 3-monthly = 0.587) [13], rather than the estimated rate from the study cohort (mean, 3-monthly = 0.345).

## Results

### Deterministic analysis

Without consideration of uncertainty in the model’s parameters, it is estimated that specialist wound clinics are a cost-effective approach to the management of chronic wounds. On average, the annual, per-patient cost of services provided via specialist clinics is $3,947 lower than for services provided as part of usual care (Table 3). The mean NMB associated with specialist clinics is positive, indicating that on average, specialist clinics are a cost-effective approach. When patient out of pocket costs are excluded and only health system related costs are included in the model, there are larger reductions in the cost to the health system. From this perspective, the average annual, per-patient cost of services provided via specialist clinics is $6,733 lower than for services provided as part of usual practice. This represents an opportunity for significant savings to be made within the system, if service provision was directed more towards specialist wound care.

**Table 3:** Deterministic results of the economic model (per patient, per year).

	Usual Care	Specialist Clinics
Out of pocket costs	$2,014	$4,802
Incremental out of pocket costs	$2,788	
Health system costs	$12,563	$5,829
Incremental health system costs	-$6,733	
Total costs	$14,573	$10,625
Total incremental costs	-$3,947	
Total QALYs	0.80	0.84
Total incremental QALYs	0.04	
Net monetary benefit	-	$6,539

### Probabilistic analysis

**Fig 2:**
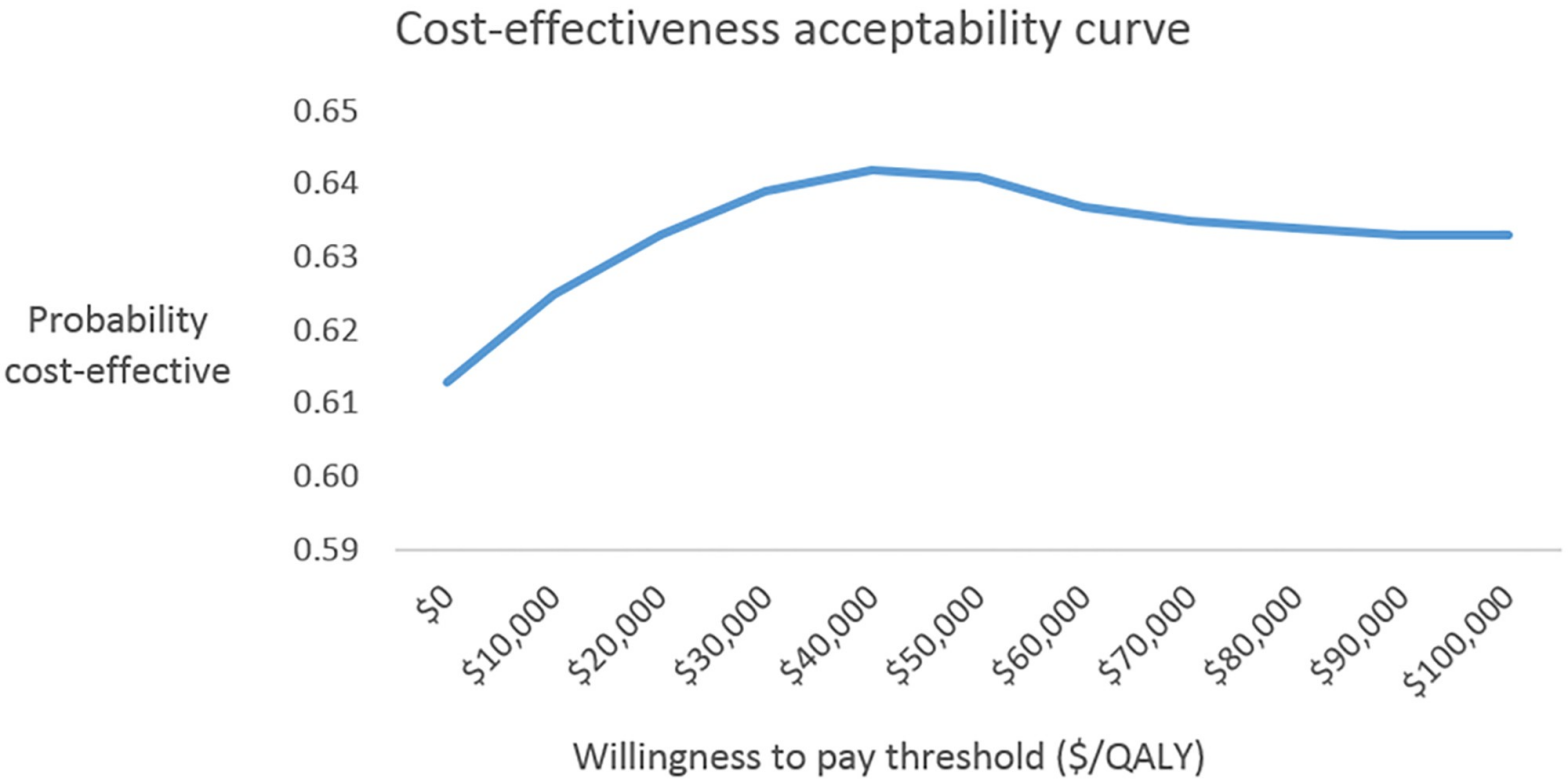
Probability that specialist clinics are cost-effective for a range of willingness to pay thresholds.

Using the net monetary benefit framework to assess cost-effectiveness, the results confirm that specialist wound clinics are a cost-effective approach to wound management. At the chosen decision-maker’s willingness to pay threshold of $64,000/QALY, specialist wound clinics are cost-effective in 64% of all simulations, meaning that for a decision-maker who is interested in maximising health gain per dollar spent, the optimal strategy is to utilise specialist clinics. The cost-effectiveness acceptability curve in Fig 2 shows that specialist clinics is cost-effective for all willingness to pay thresholds between $0/QALY and $100,000/QALY. This means that even if a decision-maker was not willing to pay anything for health gains, a threshold of $0/QALY, specialist clinics would remain the optimal strategy.

### Scenario analysis

Specialist clinics was the optimal strategy for the majority of scenarios—the solitary scenario when usual care was deemed to be optimal was when only patient out of pocket costs were included in the analysis. Even in this instance, there was high uncertainty in the results, with the probability that usual care is cost-effective being only a 50–50 outcome. There was higher certainty in all other decisions, which can be seen in Table 4. The scenario with the most certain decision was when the healing rate for specialist clinics was altered in line with previously published estimates, which was cost-effective in 78% of all 10,000 simulations.

**Table 4:** Results from probabilistic analysis, including scenario analyses.

Model	Mean NMB (Min:Max)	Optimal Strategy	Probability cost-effective
Baseline	$6,578 (-$98,546:$103,019)	Specialist Clinics	64%
Health system costs only	$9,653 (-$80,751:$153,744)	Specialist Clinics	70%
Out of pocket costs only	-$152 (-$66,199:$52,457)	Usual Clinics	51%
Lower willingness to pay for bed days	$2,080 (-$82,046:$69,299)	Specialist Clinics	56%
Altered healing rate	$13,172 (-$76,655:$121,816)	Specialist Clinics	78%

## Discussion

The focus of this evaluation has been to provide new evidence regarding the economic benefit of widespread implementation of specialist clinics for wound treatment and management in the Australian healthcare setting. This study shows that specialist clinics provide good value for money when compared to the current approach to service delivery, which is disjointed and costly. The cost savings are driven by fewer people in the specialist clinic cohort requiring hospitalisation, compared to those in the usual care cohort. Hospitalisations incur high service costs, including regular investigations such as pathology and microbiology, specialist attendances, and high costs associated with length of stay. Reducing the rate with which people require hospital-based care has a positive impact from an economic perspective.

Further, improvement in healing rates for patients who have an uncomplicated wound reduces the costs associated with seeking treatment and also contributes to the improvements in patient reported health related quality of life. Patients who attend a specialist clinic showed a higher rate of healing and lower likelihood of wound-related complications, compared to usual care. As a result, timely access to specialist care needs to be strongly encouraged, to ensure that as many health benefits are achieved as possible. These findings echo those reported elsewhere where it has been reported that utilising evidence-based practice for chronic wound treatment leads to faster healing, lower likelihood of severe infection and fewer hospital admissions [12, 31–32].

A recent Australian forum, hosted in Queensland in 2017, saw a number of key stakeholders in chronic wounds management identify a lack of financial support as a significant barrier to patients accessing specialist services. Following this forum, a key recommendation was for the Australian Government to expand subsidies through the MBS and PBS to include wound-related services, such as those provided by nurse practitioners and other specialists, as well as wound products such as compression bandages, stockings and dressings [16]. Patient out of pocket costs are higher for people accessing specialist care, and without adequate Medicare reimbursement for wound services and products, wounds will remain expensive to treat for individuals. In this study, there was evidence of ‘doubling up’ on payment for services, where some patients who were paying to attend specialist clinics were still paying for services that were provided outside of this clinic. This is unnecessary, given that specialist clinics have the capacity to stand-alone in the management and treatment of all wounds. It is clear that the benefit of specialist clinics is not yet being fully realised by patients, who could see a reduction in their out of pocket costs by reducing extra visits outside the specialist clinic. As the results of our analysis show, there is a reduction in overall costs to the health system if patients are treated in a specialist clinic setting. High patient out of pocket costs have been recognised as a common barrier to specialist clinic utilisation in previous studies published elsewhere [7,33] and if service provision was more actively promoted towards specialist clinics with adequate Medicare reimbursement for wound services and products, system-wide savings could be realised.

The focus of this evaluation has been to provide new evidence regarding the economic benefit of widespread implementation of specialist clinics for wound treatment and management in the Australian healthcare setting. With the majority of literature focusing on the evaluation of specific chronic wound treatments and their impact on patient outcomes, or the cost of care, this study provides novel economic evidence about the impact a transdisciplinary, evidence-based change in service provision has for the treatment of chronic wounds. Our results show that specialist clinics provide good value for money when compared to the current approach to service delivery, which is disjointed and costly.

### Limitations

Our study has limitations. Due to the study being conducted soon after the specialist clinic began operating and slow initial patient flow, primary data was collected from a relatively small cohort of patients who were accessing services at one specialist clinic, in one State of Australia. This means that the results should be generalised to other jurisdictions with caution. External timeframes were limited for the prospective phase of the data collection, resulting in a shortened follow up period. However transition probabilities were adjusted where appropriate to account for this.

As some data used to inform costs and transition probabilities were patient-reported, comparison with clinical records, clinical expertise and MBS/PBS data was performed to ensure accuracy. Due to the small number of participants, our study was a pre/post design rather than a randomised trial. The lack of randomisation is a limitation, but we feel our approach was valid given the availability of data, and is not uncommon [34]. Despite this, the structure of the study is based on a flexible and adaptable Markov model, allowing it to be updated as newly identified or higher quality estimates become available. As a result, the model can be run with updated data, making the results more appropriate for local decision-making in other settings.

As with all modelling studies, the results are somewhat dependent on the assumptions made regarding the parameters used for the different alternatives. In our model, we assumed that patients could not move directly between having a ‘complicated wound’ and the ‘healed’ health state, rather, that they would be required to go via the ‘uncomplicated’ health state. Further, we assumed that all patients who enter hospital for treatment have a complicated wound, although we did not differentiate between complication severities. As such, we assume that all complications have the same length of stay (LOS) and quality of life decrement, which may have resulted in an under- or over-estimation of health and cost outcomes for those patients in this health state. We also assumed that you cannot remain in the complicated health state, meaning the maximum continuous hospital LOS is one month for this model. This assumption was based on the median LOS for those patients who required hospitalisation in our study, which was 30 days, but the primary data does show a wide range in LOS (range 2–120 days). Our decision to exclude remaining in hospital may under- or over-estimate the outcomes associated with this health state for individuals, but we felt that since we were not stratifying hospital stay by complication type, and that we are presenting average results for a diverse cohort, our approach is appropriate.

Further limitations exist in the way we collected costing data. The Australian Department of Human Services (DHS) does not collect data on Commonwealth funded programs such as community nursing, outpatient clinics or public admissions to public hospitals, so costs associated with the use of these services were calculated using patient-reported information, relevant government agency websites or averages from comparable MBS and PBS data. Information about claims via the Department of Veterans’ Affairs or Repatriation Pharmaceutical Benefits Schedule are also not available via the DHS. PBS data is restricted to data recorded from prescriptions where the cost of a pharmaceutical was greater than the patient contribution, therefore some prescription costs may not have been provided. Due to the study’s timeframe and the delay in PBS claims information being included on DHS records, some DHS data has not been included for some participants. Where this occurred, comparable averages were used to estimate resource use and costs. Finally, the analysis was carried out for all chronic wound types combined, but the transition probabilities for recurrence in both the usual and specialist care cohorts came from estimates for venous leg ulcers. This approach was taken because the majority of participants in the study (73%) had venous leg ulcers.

## Conclusion

There is currently wide variation in the way that wounds-related services are managed and delivered in the Australian healthcare setting. This study shows that system-wide cost savings, as well as improvements in patient outcomes and out of pocket costs are expected if service provision is geared towards specialist clinics, particularly with increased subsidy from the Australian government. The results support further investment in specialist clinics, providing evidence that such investment is an efficient use of limited healthcare resources.

## Supporting information

S1 Appendix CHEERS checklist—Items to include when reporting economic evaluations of health interventions. (DOCX)

S2 Appendix Breakdown of costs included in the economic model. (DOCX)
