# Supplementary material for: Cost-effectiveness analysis of an innovative model of care for chronic wounds patients
Source: PLoS One. 2019 Mar 6;14(3):e0212366. doi: 10.1371/journal.pone.0212366 (PMC6402622; doi:10.1371/journal.pone.0212366)
Supplement: S2 Appendix — (DOCX) [file pone.0212366.s002.docx]

S2 Appendix: breakdown of costs included in the economic model

|  |  |  |  |
| --- | --- | --- | --- |
|  | **$/Month** | **Included** | **Excluded** |
|  | *Mean, per Px* |  |  |
| Usual Care |  |  |  |
| *‘Uncomplicated’* |  |  |  |
| Out of pocket costs | $229 | Travel; parking; consumables; private specialists | Productivity losses |
| MBS/PBS costs | $136 | Investigations; specialist or GP appointments outside hospital; wound related medications | NA |
| Commonwealth Programs | $156 | Staff wages; services covered by Commonwealth funding | NA |
|  |  |  |  |
| *‘Complicated’* |  |  |  |
| Investigations | $6,319 | In-hospital services covered under MBS; investigations | Private health insurance out of pocket costs |
| Bed day costs | $24,000 | Fixed costs | Private health insurance out of pocket costs |
|  |  |  |  |
| *‘Healed’* |  |  |  |
| VLU | $33 | Stockings (4/yr); topical cream (2/yr); quarterly GP visits | NA |
| DFU | $17 | Podiatry (4/yr); orthotic refurbishment (1/yr); orthotic replacement (1/3yrs) | NA |
| MBS costs | $41 | GP consult; podiatry consult |  |
|  |  |  |  |
| Specialist Clinic |  |  |  |
| *‘Uncomplicated’* |  |  |  |
| Out of pocket costs | $715 | Travel; parking; consumables; private specialists | Productivity losses |
| MBS/PBS costs | $248 | Investigations; specialist or GP appointments outside hospital; wound related medications | NA |
| Commonwealth Programs | $206 | Staff wages; services covered by Commonwealth funding | NA |
|  |  |  |  |
| *‘Complicated’* |  |  |  |
| Investigations | $6,319 | In-hospital services covered under MBS; investigations | Private health insurance out of pocket costs |
| Bed day costs | $24,000 | Fixed costs | Private health insurance out of pocket costs |
|  |  |  |  |
| *‘Healed’* |  |  |  |
| VLU | $92 | Stockings (4/yr); topical cream (2/yr); specialist clinic visits (4/yr) | NA |
| DFU | $73 | Podiatry (4/yr), orthotic refurbishment (1/yr); orthotic replacement (1/3yrs) | NA |
